# New treatments addressing the pathophysiology of hereditary angioedema

**DOI:** 10.1186/1476-7961-6-2

**Published:** 2008-04-14

**Authors:** Alvin E Davis

**Affiliations:** 1 Professor of Pediatrics, Harvard Medical School, Senior Investigator, Immune Disease Institute, 800 Huntington Avenue, Boston, MA 02114, USA

## Abstract

Hereditary angioedema is a serious medical condition caused by a deficiency of C1-inhibitor. The condition is the result of a defect in the gene controlling the synthesis of C1-inhibitor, which regulates the activity of a number of plasma cascade systems. Although the prevalence of hereditary angioedema is low – between 1:10,000 to 1:50,000 – the condition can result in considerable pain, debilitation, reduced quality of life, and even death in those afflicted. Hereditary angioedema presents clinically as cutaneous swelling of the extremities, face, genitals, and trunk, or painful swelling of the gastrointestinal mucosa. Angioedema of the upper airways is extremely serious and has resulted in death by asphyxiation.

Subnormal levels of C1-inhibitor are associated with the inappropriate activation of a number of pathways – including, in particular, the complement and contact systems, and to some extent, the fibrinolysis and coagulation systems.

Current findings indicate bradykinin, a product of contact system activation, as the primary mediator of angioedema in patients with C1-inhibitor deficiency. However, other systems may play a role in bradykinin's rapid and excessive generation by depleting available levels of C1-inhibitor.

There are currently no effective therapies in the United States to treat acute attacks of hereditary angioedema, and currently available agents used to treat hereditary angioedema prophylactically are suboptimal. Five new agents are, however, in Phase III development. Three of these agents replace C1-inhibitor, directly addressing the underlying cause of hereditary angioedema and re-establishing regulatory control of all pathways and proteases involved in its pathogenesis. These agents include a nano-filtered C1-inhibitor replacement therapy, a pasteurized C1-inhibitor, and a recombinant C1-inhibitor isolated from the milk of transgenic rabbits. All C1-inhibitors are being investigated for acute angioedema attacks; the nano-filtered C1-inhibitor is also being investigated for prophylaxis of attacks. The other two agents, a kallikrein inhibitor and a bradykinin receptor-2 antagonist, target contact system components that are mediators of vascular permeability. These mediators are formed by contact system activation as a result of C1-inhibitor consumption.

## Review

Hereditary angioedema (HAE) is an autosomal dominant condition caused by mutations to the gene controlling C1-inhibitor production. This gene would seem to be relatively mutable. As many as 25% of new patients have no family history and presumably represent new mutations. In addition, over 150 different mutations have been identified [1-3]. Most of the identified mutations have been included in a C1-inhibitor gene mutation database [4]. Although the exact prevalence of HAE is unknown, it has been estimated that the condition affects between 1 in 10,000 to 1 in 100,000 individuals [5-7]. HAE was first clinically described by Heinrich Quincke, in 1882. Virginia Donaldson and colleagues, about 75 years later, identified the biochemical defect leading to HAE as subnormal or ineffective levels of C1-inhibitor. C1-inhibitor regulates the activity of the first component of the complement system, C1-esterase, controlling both C1's rate of activation, as well as deactivating activated C1. C1-inhibitor is also able to inactivate a number of other proteases in other plasma cascade systems [1,3,8].

Specific mutations have resulted in two main types of HAE. Type 1 (accounting for approximately 85% of HAE patients) is characterized by subnormal levels of circulating

C1-inhibitor. Given the heterozygous nature of the condition, it might be presumed that plasma levels of C1-inhibitor in individuals with the mutation would be 50% of normal. In fact, levels are typically much lower – between 5% and 30% [2,3]. These low levels suggest enhanced depletion of C1-inhibitor – the rate of consumption exceeding the rate of ongoing synthesis – in patients with the genetic defect, even during asymptomatic periods [9]. In Type 2 HAE (approximately 15% of patients), C1-inhibitor plasma levels are normal or elevated. High concentrations of the mutant protein are typically present due to the increased half-life of the dysfunctional C1-inhibitor, which fails to form inhibitor-protease complexes. Differences in disease severity, manifestation, or clinical course have not been associated with HAE type, but both types are associated with a deficiency in functional C1-inhibitor [2,3].

## Clinical Presentation

Increased levels of vascular permeability factors associated with C1-inhibitor deficiency may result in sudden local diminishments of endothelial barrier function. Plasma may then leak from capillaries deeper into cutaneous or mucosal tissue layers [1,8]. HAE-associated swelling typically occurs in the facial area and extremities, the upper airways, the genitourinary tract, and in the gastrointestinal mucosa. Far less frequent though also reported are episodes involving the soft palate, the tongue, urinary bladder, chest, muscles, joints, kidneys, and the esophagus [10]. Cutaneous edema is debilitating, may be painful, and can severely affect quality of life. Abdominal angioedema can be extremely painful, severe enough to cause gastrointestinal tract obstruction, and is often accompanied by diarrhea and/or vomiting [1,2,8,11]. In a retrospective assessment of 33,671 abdominal angioedema attacks in 153 patients, Bork and colleagues reported a mean maximal pain score of 8.4 (range 1–10). Vomiting accompanied 71% of the attacks, and diarrhea 41%. Circulatory collapse and loss of consciousness were also described [12]. Abdominal angioedema is often mistaken for a surgical emergency; as many as 1/3 of patients with undiagnosed HAE have undergone exploratory laparotomy or appendectomy during abdominal attacks [13].

The most serious form of HAE affects the upper airways and involves swelling of the larynx and pharynx. Prior to the development of effective diagnostic techniques and acute care interventions (where they are available) as many as 40% of patients with HAE died from an episode of laryngeal edema. Bork and associates have also reported a mortality rate as high as 50% associated with laryngeal edema in patients with undiagnosed HAE [14,15]. Frequency of attacks and age of onset may show considerable variation, and the pattern of attacks may change with age. Attacks typically involve a single site, though simultaneous attacks at multiple sites are not uncommon [1].

### C1-inhibitor

C1-inhibitor is a protein whose biological function is to inhibit a number of other proteases involved in the response to infection, injury, or inflammation. C1-inhibitor is the primary regulator of contact and complement system activation, and may play a minor role in the regulation of coagulation and fibrinolysis [2,3,16,17]. Inappropriate activation of these plasma pathways, particularly of the complement and contact systems, as a result of C1-inhibitor deficiency, is a central component in the pathophysiology of HAE [1,2,8,18,19]. C1-inhibitor inactivates C1r and C1s, the serine protease subcomponents of the first component of the complement pathway [20]. C1-inhibitor also may play a minor role in the regulation of the coagulation cascade by means of its inhibitory effects on factor XIIa and factor XIa, as well as on thrombin formation [1,2,18,20,21]. In the fibrinolytic pathway, C1-inhibitor participates in the inactivation of plasmin and tissue plasminogen activator (tPA). However, under normal physiologic conditions, C1-inhibitor is not an important inhibitor of either of these proteases [1,2,19,20,22]. In the contact system pathway, C1-inhibitor inactivates both factor XIIa and active kallikrein, thereby preventing both the activation of kallikrein from prekallikrein and the formation of bradykinin, a vascular permeability factor [2,20,21,23-25]. Given its regulatory effects on kallikrein, C1-inhibtor might well have been designated "kallikrein inhibitor."

### Mediators of Vascular Permeability

Although C1-inhibitor deficiency has been known to be the underlying cause of HAE for more than 40 years, the actual mediator(s) of the vascular permeability characteristic of the disease remains the subject of continued investigation. Because the subcutaneous edema associated with HAE is often painless, and because subcutaneous injections of bradykinin are acutely painful, investigators initially believed that a complement-derived permeability factor would be the most likely mediator of the angioedema associated with C1-inhibitor deficiency [2,20]. A complement-derived substance, designated C2 kinin, was initially proposed as a candidate permeability mediator, but subsequent investigations failed to verify its activity [26-28].

Bradykinin, a nonapeptide released from kininogen by kallikrein cleavage, is a downstream product of contact system activation. It is capable of inducing edema as a result of its effects on vasodilation and microvessel permeability [29]. In vivo investigations demonstrated rapid elevations in bradykinin in C1-inhibitor-deficient patients during HAE attacks [30,31]. However, the strong linkage of bradykinin and angioedema attacks does not preclude involvement of other plasma cascade products, such as plasmin and thrombin, in the initiation and duration of HAE attacks. Clinical and experimental data have indicated that thrombin formation in the coagulation pathway is increased during HAE attacks [18]. Several lines of evidence suggest that plasmin and the fibrinolytic pathway may also have some involvement in HAE [2,19].

### Pathogenesis of HAE Attacks

The main pathogenic mechanism for the generation of HAE attacks is depletion and/or consumption of C1-inhibitor. Clinically, attacks of HAE appear to have a number of environmental and pathophysiological triggers: eg, prolonged mechanical pressure, trauma, emotional stress, menses, or intercurrent illness, particularly inflammation [1,8]. Angiotensin converting enzyme inhibitor therapy may trigger attacks in individuals with HAE [32]. In persons with a mutation associated with C1-inhibitor deficiency, angioedema attacks may occur spontaneously even in the absence of an overt precipitating factor. Chronically low levels of C1-inhibitor – ≤ 30% of normal – suggest the possibility of complement and contact systems activation even during apparently symptom-free periods, so-called autoactivation of the plasma cascade systems. Any further reductions in available C1-inhibitor would be associated with development of angioedema symptoms [2].

Since C1-inhibitor is a primary regulator of a number of proteases and pathways, the activation of any of these proteases and pathways could also lead to further consumption of C1-inhibitor and the development of HAE symptoms. Chronic, low-level activation of the complement pathway could lead to the inappropriate activation of the contact pathway. Vascular permeability and edema would result from the rapid and excessive release of bradykinin [1,20]. Cugno and colleagues have speculated that the significant increases in prothrombin fragment F1+2 in the coagulation pathway may involve increased plasma levels of factor XII, an initiator of the contact pathway that is activated during HAE attacks [18]. In addition, factor XIIa and plasmin may serve to activate C1 in the complement pathway, while factor XIIa or kallikrein in the contact pathway may generate plasmin from plasminogen in the fibrinolytic pathway [20].

While it may be that only the contact system and bradykinin are directly implicated in the release of vascular permeability mediators and angioedema, activation of other plasma systems, particularly the complement system, may contribute to the genesis, severity, and duration of the attack by contributing to the consumption of C1-inhibitor. Activation of these other pathways may also contribute proteases and factors that could play a role in HAE attacks. These processes result in a sequence of C1-inhibitor consumption, complement activation, and release of bradykinin during every acute attack until appropriate therapy is administered to raise serum levels of C1-inhibitor, or until remission spontaneously occurs [1,8].

### Therapies for the Management of HAE

Since no effective therapies for acute HAE attacks are available in the United States, treatment is suboptimal, and may often result in significant medical, emotional, and economic consequences. Frequent hospitalizations and surgical procedures have been associated with this condition, particularly in untreated or inadequately treated patients. In the case of life-threatening laryngeal angioedema, intubation and tracheotomy have been indicated. Inaccurate diagnosis of HAE has resulted in unnecessary surgeries and other medical procedures [1,8,10,32].

As with acute therapy, currently available HAE prophylactic treatment options in the U.S. are suboptimal. Attenuated androgens, particularly danazol and stanozolol, have been used for decades, with good efficacy – while these agents do not prevent all attacks, they do reduce the number. Long-term use of these agents, however, is associated with substantial risk of side effects and adverse events, including weight gain, viralization and menstrual irregularities in women, and dyslipidemia [1,8,33-36]. Szeplaki and colleagues, who found long-term danazol therapy to be associated with the development of unfavorable lipid profiles, concluded that long-term danazol prophylaxis should be considered a significant risk factor for atherosclerosis in patients with HAE, a risk that would be compounded in patients also experiencing blood pressure elevations as a result of danazol therapy [36].

In a long-term assessment of HAE prophylaxis with attenuated androgens (median treatment time:125.5 months), Cicardi and colleagues noted an apparent association between androgen therapy and incidence of arterial hypertension. While only a single untreated patient (3%) developed hypertension during the study period, nine danazol-treated patients (25%; age range, 35 to 74 years, median age 60) developed hypertension – in some cases within a few months of therapy initiation [33]. Hypertension was also found to be a significant adverse event in a long-term study of danazol prophylaxis in women with HAE (mean age 35.2 years, mean duration of therapy 60 months). In this study 10% of patients (6/60) developed hypertension [37]. Salt and water retention associated with danazol therapy may explain both the weight gain and hypertension observed in some patients.

Long-term administration of attenuated androgens has been associated with a number of liver disorders, including hepatic cell necrosis and cholestasis [1,38,39]. There have been case reports of a number of instances of hepatotoxicity associated with long-term danazol therapy, including hepatocellular adenoma and hepatocellular carcinoma. Bork and colleagues have described four cases of hepatocellular adenoma associated with long-term (> 10 yrs) danazol prophylaxis for HAE [40,41]. Several cases of hepatocellular carcinoma associated with long-term danazol therapy have also been reported, although in these instances the patients were not being treated for HAE (ie, a female patient with systemic lupus erythematosus treated for 4 years with danazol; and a female patient with idiopathic thrombocytopenia purpura refractory to corticotherapy, intravenous immunoglobulins, vincristine, and splenectomy, treated with 600 mg danazol daily for 5 years) [42,43].

It is also of concern that the prevalence and severity of adverse effects associated with attenuated androgens appear to increase with dosage strength and duration of therapy [36,44,45].

The lack of therapeutic options should soon be remedied. Five new therapies are in Phase III clinical development: a kallikrein inhibitor (DX-88), a bradykinin receptor-2 antagonist (Icatibant), and three C1-inhibitor replacement therapies.

Designed by phage display technology, DX-88 is a recombinant protein capable of binding to and inhibiting human kallikrein. It has a plasma half-life of approximately 70 minutes when administered intravenously (IV) and 2 hours when administered subcutaneously (SC). It has been evaluated for safety and efficacy in several trials at a range of doses (eg, 5, 10, 20, or 40 mg/m^2^, given intravenously). Patients have reported significant symptom improvement versus placebo. Serious adverse events have been reported in a small number of patients, including shortness of breath and throat edema, as well as prolonged prothrombin and thrombin in one patient. Four patients were observed with post-treatment activated partial thromboplastin times considered abnormal by the investigator [1,46-48].

Icatibant, a bradykinin receptor-2 antagonist is a synthetic decapeptide with a structure similar to bradykinin; it is a highly specific antagonist for bradykinin receptor-2, with a plasma half-life of approximately 2–4 hours [49]. In an uncontrolled pilot study, 15 patients (with 20 HAE attacks) were treated with one of five dosage strengths of Icatibant (three IV doses: 0.4 mg/kg body weight administered IV over a period of 2 h; 0.4 mg/kg administered over a period of 0.5 h; 0.8 mg/kg administered over a period of 0.5 h; or two SC doses: 30 mg SC; 45 mg SC). Compared with untreated attacks, Icatibant reduced the mean time to onset of symptom relief by 97%, from 42 ± 14 hours to 1.16 ± 0.95 hours for all dosage groups. However, relapse might be an issue. Four patients experienced five attacks subsequent to treatment (between 14 hours and 27 hours). The 5 attacks were successfully treated with rescue C1-inhibitor (Berinert P (1000 U or 500 U). All patients in whom attacks recurred showed initial response to Icatibant, including symptom relief [49].

Three C1-inhibitor replacement products are also in Phase III development: a pasteurized C1-inhibitor, Berinert P, with a plasma half-life of between 32 and 47 hours [50]; a recombinant human C1-inhibitor isolated from the milk of transgenic rabbits, rhC1INH (Rhucin), with a plasma half-life of ~3 hours [1,51]; and a nano-filtered C1-inhibitor, Cinryze (pharmacodynamics/pharmacokinetic data not yet available; Cetor, a comparable agent though lacking the nano-filtration process in its preparation, has a half-life of 48 ± 10 hours)[52]. Nano-filtration is a purification process that has a number of efficient and robust steps for both virus inactivation or removal and prion removal [53]. C1-inhibitor replacement therapy not only suppresses bradykinin release by inactivation of factor XIIa and kallikrein, but also suppresses activation of the complement system and perhaps of the fibrinolytic and coagulation pathways. Although unproven, it is possible that ongoing activation of these pathways indirectly contributes to the contact system activation via two mechanisms. First, activation of proteases susceptible to inactivation by C1-inhibitor would result in depletion of C1-inhibitor. Complement system activation, in particular, would deplete C1-inhibitor because C1r and C1s are present in greater quantities than most of the other proteases and because complement activation in HAE tends to be extensive. Secondly, a number of in vitro experiments have suggested, as described previously, that there may be interactions among the contact, complement and fibrinolytic systems in which a protease in one system directly activates a protease in one or both of the other systems. If such interactions take place in vivo, activation of one system could eventuate in activation of all three systems. This would result in release of bradykinin via contact system activation and would further enhance C1-inhibitor consumption. However, it must be emphasized that these interactions have not been shown to occur *in vivo*.

By inhibiting all of the susceptible proteases of the complement, contact, and fibrinolytic pathways, purified C1-inhibitor replacement therapy may turn out to provide more efficient control of angioedema symptoms. However, this assumption remains to be proven. A related issue is the observation that some patients, following treatment, develop recurrent attacks of angioedema after 24 – 48 hours. It is possible that these recurrent attacks are a function of the half-life of the therapeutic agent or they could be related to the absence of inhibition of all the proteases susceptible to C1-inhibitor. Long-term recovery from an attack presumably requires the stabilization of levels of C1-inhibitor that are sufficiently high to prevent a recurrence of significant contact system activation with resultant bradykinin release. If activation of both the contact and complement systems is suppressed, C1-inhibitor levels might recover more rapidly and early recurrences might be suppressed. Because plasma-derived C1-inhibitor has a longer half-life and is a broader spectrum inhibitor, it has been assumed that such recurrences occur less frequently with C1-inhibitor therapy but that assumption awaits verification.

C1-inhibitor has been available for decades in Europe where it has compiled considerable clinical efficacy and safety data. In the United States, Waytes and colleagues treated 11 patients experiencing a total of 55 HAE attacks with vapor-heated C1-inhibitor concentrate and 11 patients experiencing 49 HAE attacks with placebo [54]. Nearly all HAE attacks (95%) treated with C1-inhibitor responded to treatment, with an average symptom improvement response time of ~55 minutes, compared with just 12% of placebo-treated attacks (P < 0.001). No adverse events were associated with C1-inhibitor concentrate treatment. As mentioned, three C1-inhibitor products are undergoing or have completed Phase III development in the U.S.

Where it has been available, C1-inhibitor concentrate purified from human plasma has also been used effectively as a long-term prophylaxis for HAE attacks [1]. Waytes and colleagues reported > 60% reduction in disease activity in patients treated prophylactically with C1-inhibitor. Treatment consisted of 5 infusions of either C1-inhibitor or placebo every third day over two 17-day periods separated by at least 3 weeks. The second study period alternated the treatments. No patient receiving C1-inhibitor concentrate demonstrated objective signs of either laryngeal or genitourinary edema, whereas 4 of 6 placebo-treated patients demonstrated evidence of attacks in one or both of those systems [54].

Additionally, in a small study of patients self-administering C1-inhibitor concentrate, 12 patients in the prophylactic group (10 patients with hereditary C1-inhibitor deficiency and 2 patients with acquired C1-inhibitor deficiency; there were also 31 patients in the on-demand group) experienced an attack rate reduction from a mean of 4 attacks per month to 0.3 attacks per month. The mean interval between prophylactic injections was 6.8 ± 1.0 days. The mean follow-up time for these patients was 3.5 years [55]. In the United States, one of the C1-inhibitors currently in development, the nano-filtered agent, cinryze, is also seeking an indication for prophylaxis in addition to an indication for acute attack treatment.

Whether or not a patient with HAE requires a prophylaxis regimen will upon a number of patient selection criteria, including frequency and severity of attacks, and the site of attacks. Data concerning the number or percentage of HAE patients either receiving prophylaxis therapy, or who might be candidates for prophylaxis, are sparse. In their review of clinical experience of 235 HAE patients over a period of 19 years, Agostoni and Cicardi found that 30% experienced more than 1 attack per month; these patients were considered candidates for continuous prophylactic treatment [13]. A Spanish registry study of 444 patients with HAE found that treating physicians considered approximately 85% of those patients to be symptomatic. Of those patients, 63% received long-term prophylaxis, although the criteria upon which prophylaxis was recommended (by physicians) and accepted (by patients) were not specified [6]. As stated, the decision to recommend, and to accept, HAE prophylaxis should be based upon a number of criteria including symptom severity, side effects' concerns, risk and impairment, etc. Therapy should always be individually tailored to meet specific patient needs and requirements.

## Conclusion

Hereditary angioedema is a genetic disorder whose underlying cause is a deficiency of C1-inhibitor. Although prevalence is relatively low, the disease can result in significant morbidity and mortality for those afflicted. The goals of HAE therapy are disease management – ie, preventing attacks, ideally by re-establishing normal physiology, and improving quality of life; and crisis management – ie, treating acute attacks with utmost efficacy, rapidity, and safety. Several new therapies for HAE are in development. The kallikrein inhibitor and the bradykinin receptor-2 antagonist target biologically active products of contact pathway dysregulation caused by C1-inhibitor deficiency. These agents inhibit the release or block the activity of bradykinin, the primary mediator of vascular permeability associated with HAE. They do not address the primary pathophysiologic cause of HAE – C1-inhibitor deficiency. Several C1-inhibitor replacement products are in development, including a pasteurized product, a transgenic agent, and a nano-filtered C1-inhibitor concentrate. C1-inhibitor replacement therapy addresses the primary cause of HAE by replacing C1-inhibitor. C1-inhibitor products have been available for decades in Europe, where they have been the treatment of choice for acute attacks. C1-inhibitor concentrate restores regulatory control over all pathways and biologically active products that may play a role, either directly or indirectly, in the pathogenesis of HAE.

## Abbreviations

Hereditary angioedema, HAE; intravenous, IV; subcutaneous, SC.

## Competing interests

*Financial Competing Interests*: In the past five years, the author has received reimbursements and consulting fees from each of the following companies: CSL Behring, Dyax, Jerini, Lev Pharmaceuticals, and Pharming Group NV. Lev Pharmaceuticals provided funds for the article-processing charge for this manuscript.
